# Nanocrystalline (AlTiVCr)N Multi-Component Nitride Thin Films with Superior Mechanical Performance

**DOI:** 10.3390/nano12152722

**Published:** 2022-08-08

**Authors:** Chuangshi Feng, Xiaobin Feng, Zhou Guan, Hongquan Song, Tianli Wang, Weibing Liao, Yang Lu, Fuxiang Zhang

**Affiliations:** 1 Songshan Lake Materials Laboratory, Dongguan 523808, China; 2 Hubei Key Laboratory of Theory and Application of Advanced Materials Mechanics, Wuhan University of Technology, Wuhan 430070, China; 3 Department of Mechanical Engineering, City University of Hong Kong, Hong Kong 999077, China; 4 College of Physics and Optoelectronic Engineering, Shenzhen University, Shenzhen 518060, China; 5 Nano-Manufacturing Laboratory (NML), City University of Hong Kong Shenzhen Research Institute, Shenzhen 518060, China

**Keywords:** multi-component alloy, nitride thin film, nanocrystalline, in situ compressive test

## Abstract

Multi-component nitride thin films usually show high hardness and good wear resistance due to the nanoscale structure and solid-solution strengthening effect. However, the state of N atoms in the thin film and its effects on the compressive strength is still unclear. In this work, (AlTiVCr)N multi-component nitride thin films with a face-centered cubic (FCC) structure prepared by the direct current magnetron sputtering method exhibit a superior strength of ~4.5 GPa and final fracture at a strain of ~5.0%. The excellent mechanical properties are attributed to the synergistic effects of the nanocrystalline structure, covalent bonding between N and metal atoms, and interstitial strengthening. Our results could provide an intensive understanding of the relationship between microstructure and mechanical performances for multi-component nitride thin films, which may promote their applications in micro- and nano-devices.

## 1. Introduction

After decades of research, the preparation and mechanical properties of multi-component alloy thin films have attracted more and more attention. Many studies have shown that multi-component alloy thin films exhibit significant mechanical properties compared with the corresponding bulk alloys due to the existence of nanostructures [1,2,3,4]. To further excavate the application potential of the multi-component alloy thin films as a protective layer, reactive direct current (DC) magnetron sputtering was used to fabricate some oxide films and nitride films. Yeh et al. designed and prepared two multi-component nitride films with compositions of (Al_23.1_Cr_30.8_Nb_7.7_Si_7.7_Ti_30.7_)N_50_ and (Al_29.1_Cr_30.8_Nb_11.2_Si_7.7_Ti_21.2_)N_50_ with a face-centered cubic structure [5]. The films’ highest hardness reaches 36.1 GPa and 36.7 GPa, respectively, which can be tuned by the residual stress, crystallite size, and film densification. Pogrebnjak et al. have investigated the influence of nitrogen pressure on the fabrication of the two-phase (face-centered cubic and body-centered cubic structure) superhard nanocomposite (TiZrNbAlYCr)N coatings. The results show that the maximum hardness of the deposited coatings at 0.5 Pa nitrogen pressure was about 47 GPa [6]. The influence of deposition parameters, especially the temperature and substrate bias, on the physicomechanical properties of some nitride coatings [7] was also studied. Zaid prepared a 111-oriented, B1-structured multi-component alloy nitride (VNbTaMoW)N/Al_2_O_3_(0001) thin film using reactive magnetron sputtering with elastic modulus E of 187 ± 70 GPa, and the results suggest that the cocktail effects could enhance the stiffness of the thin film [8]. As potential engineering materials, multi-component nitride thin films have attracted enormous research interest. However, the relationship between the atomic-scale structure and the properties is unclear, such as the binding states of nitrogen and metal elements. In general, the existence of small atoms as interstitials can enhance the mechanical properties of solid solution alloys. For example, Lei et al. explored the effect of N atoms in TiZrHfNb refractory multi-component alloys, and incorporating N atoms can improve the strength and plasticity of the alloy simultaneously [9]. On the other hand, N is a nonmetallic element with a high electronegativity that can easily gain electrons from metals and form covalent bonds. The effect of the covalent and metal bonds on the mechanical properties of the multi-component nitride thin films is worth further exploration.

Lightweight structural materials exhibit great potential in engineering applications. The AlTiVCr multi-component alloy has a low density of 5.06 g cm^−3^ and a BCC structure at room temperature, which transforms to a BCC_B2 structure at low temperature [10]. Furthermore, the microalloyed AlTiVCr has a density close to 4.5 g cm^−3^ and hardness up to 710 HV by introducing nonmetallic elements [11]. It can be seen that AlTiVCr alloy has the characteristics of low density and high hardness. It is interesting to prepare the corresponding multi-component alloy films to meet engineering needs. In this work, we prepared (AlTiVCr)N multi-component nitride thin films with the sputtering method. We studied the atomic-scale structures and the relationship between the structure and comprehensive mechanical properties.

## 2. Experimental Methods

Reactive direct current (DC) magnetron sputtering with a mixture of high purity 99.99% Ar plasma and N_2_ reactive gas was used to deposit the (AlTiVCr)N thin films on the Si substrate. The ratio of the gas flow rate of N_2_ and Ar was 2.5:27.5 standard cubic centimeters per minute (sccm). The deposition was performed under a DC power of 100 W with a working pressure of 0.5 Pa, and deposition time was maintained at 2 h. The microstructure of the thin film was investigated by scanning electron microscopy (SEM; Supra 55 Sapphire, Carl ZEISS, Baden-Württemberg, Germany), and the surface roughness and the 3D surface topography were measured by atomic force microscopy (AFM; Dimension ICON, Bruker, BW, Germany). Electron probe microanalysis (EPMA; Nordly max3, Oxford, UK) was used to analyze the chemical composition. X-ray photoelectron spectroscopy (XPS; ESCALAB Xi+, Thermo Fisher, Waltham, MA, USA) was used to determine the chemical state of elements in the near-surface region of the films. The films’ hardness and elastic modulus were measured using nanoindentation (Tl-950, Hysitron, Billerica, MA, USA) with a Berkovich diamond indenter tip. The in situ compression test was fabricated and characterized by FEI Scios focused ion beam (FIB; Thermo Scientific, Waltham, MA, USA) and picoIndenter (PI 85, Hysitron, Billerica, MA, USA). The nanopillar’s diameter was kept close to half the pillar’s height, and the engineering stress and strain were calculated. The curve was tested four times to obtain reliable results.

## 3. Results and Discussion

**Figure 1:**
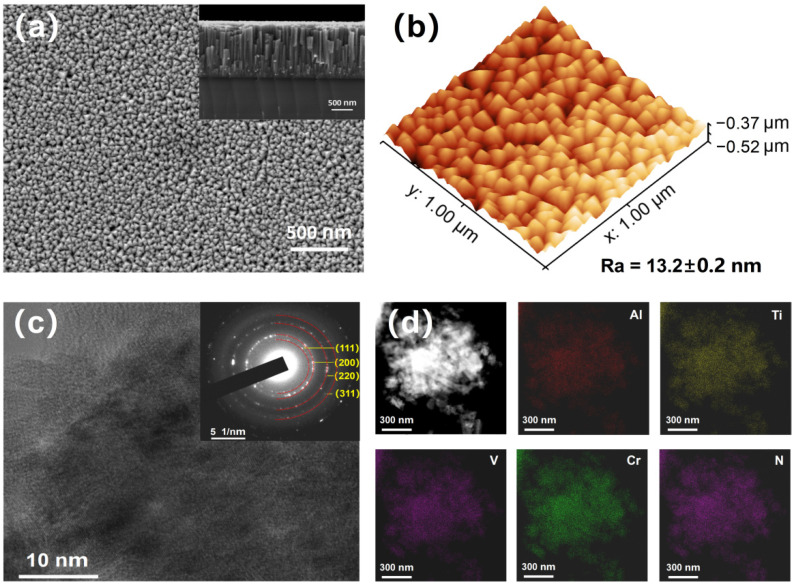
(**a**) SEM surface and corresponding cross-section morphology of the thin film; (**b**) 3D surface morphology; (**c**) HRTEM image and the corresponding SAED pattern; (**d**) EDS mapping results acquired by TEM.

The SEM surface morphology and the corresponding cross-sectional morphology of the thin film are displayed in Figure 1a. It is obvious that the thin film is homogeneous and dense with ~50 nm nano-sized triangular grains. The thickness is about 1.4 μm, and the film shows typical columnar crystallites. The nanostructured film surface is smooth with a surface height fluctuation of 0.15 μm and surface mean roughness (Ra) of 13.2 ± 0.2 nm, as shown in Figure 1b. The film exhibits a typical nanocrystalline structure, and selected area electron diffraction (SAED) pattern analysis suggests that the film is in an FCC phase structure, as shown in Figure 1c. As mentioned above, the AlTiVCr multi-component alloy exhibits a BCC structure in bulk, the formation of the FCC structure may be due to the interaction of N atoms with metal atoms and forming of covalent bonds. Although AlN shows an HCP structure, TiN, VN, and CrN all show FCC structures [12,13,14], and the final crystal structure of the film agrees with that of the majority. Figure 1d shows the EDS mapping of the multi-component nitride thin film acquired by TEM. The five elements are evenly distributed, and no segregation is found, which verifies the superiority of magnetron sputtering in preparing homogeneous materials. In order to improve the accuracy of N element content, EPMA was used to analyze the chemical composition, and the elements percentages of Al, Ti, V, Cr, N are 12.5, 9.4, 10.5, 14.1, 53.5 at.% respectively. Four metal elements of Al, Ti, V, and Cr exhibit a near equiatomic ratio, and the concentration of Al and Cr is slightly higher due to the higher sputtering yield [12]. This phenomenon can provide a reference for subsequent target composition design. It should be mentioned that the concentration of N is higher than all the metallic elements since N has larger electronegativity (3.04) than that of metal elements (electronegativity Al:1.61, Ti:1.54, V:1.63, Cr:1.66), nitrogen atoms in the film can gain electrons from all the metals and form covalent bonds.

**Figure 2:**
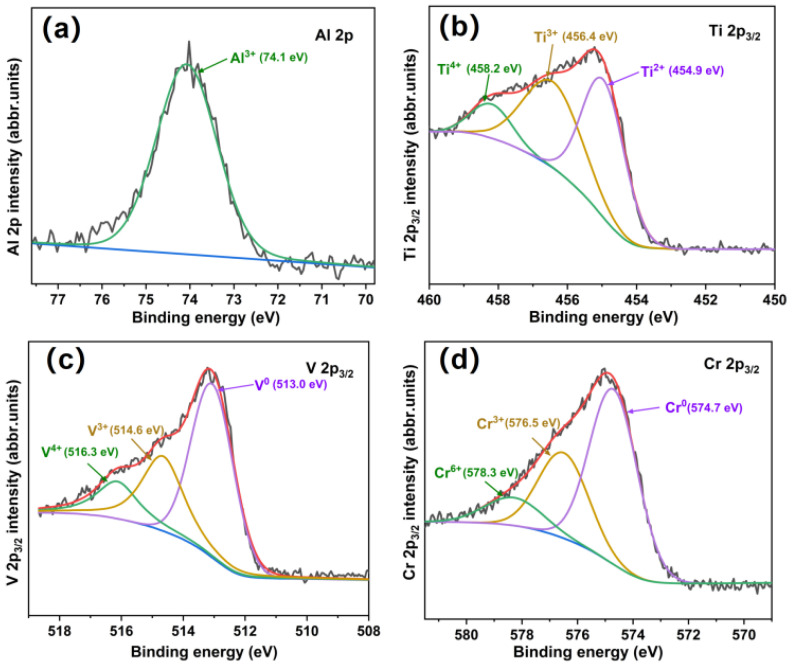
XPS spectra of Al 2p, Ti 2p3/2, V 2p3/2, and Cr 2p3/2 recorded from the outmost surface of the thin film.

In order to identify the bonding states of elements in the films, XPS measurements for all the 2p electrons of the metals were performed, and the results are illustrated in Figure 2. The deconvoluted Al 2p profile in Figure 2a exhibits only one peak at 74.1 eV, which corresponds to the valence state of Al^3+^ [15]. The Ti 2p_3/2_ spectrum is composed of three peaks centered at 454.9 eV, 456.4 eV, 458.2 eV, which represent the existence of Ti^2+^, Ti^3+^, Ti^4+^ respectively [16,17,18]. However, as shown in Figure 2c, the V 2p_3/2_ spectrum is decomposed into three peaks with center positions of 513.0 eV, 514.6 eV, and 516.3 eV, representing the existence of V^0^, V^3+^, V^4+^, respectively [19,20,21]. The existence of metallic peak V^0^ indicates that the metal atoms in the film do not completely form intermetallic compounds with N. Similarly, the Cr 2p_3/2_ spectrum in Figure 2d has a metallic peak Cr^0^ at 574.7 eV and two valence states of Cr^3+^, Cr^6+^ at 576.5 eV and 578.3 eV, respectively [22,23,24]. XPS results suggest that not all the metal elements in the film react with nitrogen atoms to form covalent bonds. The film may contain both FCC solid solution phase with N as interstitials and metallic nitrides. This may also be why the film has an FCC phase structure.

**Figure 3:**
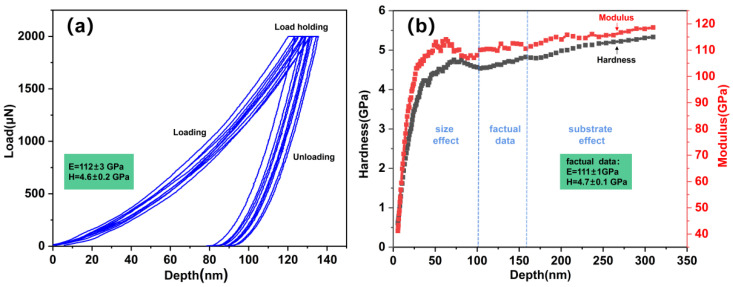
(**a**) Loading and unloading curves under nine experiments; (**b**) hardness (black line) and elastic modulus (red line) measured by continuous stiffness method.

Figure 3 shows the hardness and modulus measured with nanoindentation. The loading and unloading curves under nine experiments are presented in Figure 3a. The indentation depth was controlled at less than one-tenth of the film to exclude the effects of the substrate. The Oliver-Pharr method was used to calculate the hardness H and Young’s modulus [25]. The calculated hardness and elastic modulus are 4.6 ± 0.2 GPa and 112 ± 3 GPa, respectively. In order to avoid the influence of the surface and the substrate on the hardness and modulus of the thin film, the continuous stiffness method was used to analyze the factual data shown in Figure 3b. The derived values increase with the indentation depth when the indentation depth exceeds 160 nm, showing a large fluctuation if the indentation depth is less than 100 nm. The indentation depth of 100–160 nm is optimal to be used to measure the thin films’ factual hardness and elastic modulus. The calculated values were 4.7 ± 0.1 GPa and 111 ± 1 GPa, respectively. The selection of this range verified the reliability of loading and unloading curves and provided an effective reference for obtaining reliable nanoindentation data. The investigation of the microstructure and the elements’ bonding states suggested that the film’s high hardness may be attributed to the nanoscale grains and the nitrides in the film. As reported, the modulus depends on the atomic bonding force [26].

**Figure 4:**
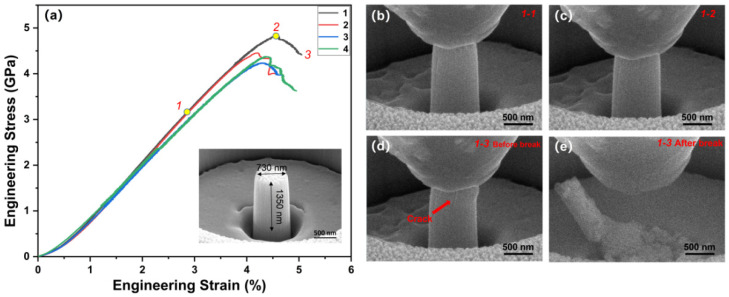
(**a**) In situ compressive stress-strain curves and the nanopillar of the number 1 curve (insertion: 730 nm in width and 1350 nm in length); (**b**–**e**) the compression morphology under different strain conditions.

The in situ compressive stress-strain curves and the compression morphology under different strain conditions of curve 1 are plotted in Figure 4. A pillar sample was prepared along the film thickness direction. To ensure the reliability of the experiments, the compression test was repeated four times as shown in Figure 4a. The calculated average modulus is around 107 ± 3 GPa, slightly lower than the nanoindentation value. Accordingly, the superior ultimate strength of the films is 4.5 ± 0.2 GPa and final fracture at a strain of ~5.0%. In order to observe the shape changes of the pillar during compression, the morphology corresponding to curve 1 is shown in Figure 4b–e. Figure 4b presents that the pillar extents a pure elastic deformation. After that, the pillar enters the elastic-plastic deformation stage under increasing stress, and the strength continues to rise to the highest in Figure 4c. In this section, the plastic deformation was not clearly observed; elastic deformation still dominates. After passing through the highest point, the curve shows a downward trend, and the pillar has an obvious plastic deformation. Moreover, cracks appeared, as shown in Figure 4d. When the strain reaches 5.0% (Figure 4e), the pillar breaks suddenly, illustrating that the deformation capacity of the pillar reaches the limit.

**Figure 5:**
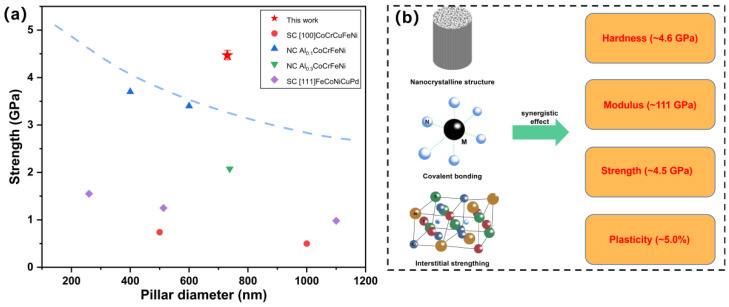
(**a**) Comparison of the strength and pillar diameter for other non-nitride multi-component alloy materials; (**b**) Schematic illustration of the microstructure and mechanical properties (M means the Al/Ti/V/Cr atoms).

It can be seen from the stress-strain curve that the pillar exhibits an elastic-plastic deformation stage before the brittle fracture and the total strain is about 5.0%. Combined with the above analysis, the reasons can be summarized as follows. Firstly, the elastic-plastic deformation behavior may come from the FCC structure, metal atoms, and interstitial N atom in the thin film, which is conducive to dislocation slip. Secondly, the complex interactions between covalent bonds, phase structures, and metal atoms lead to elastic deformation of about 3.0%. The grain boundaries in nanocrystalline can disperse deformation and hinder the propagation of cracks, thereby improving elasticity and plasticity. Thirdly, cracking and buckling of the inter columnar grains can also make additional contributions to ductility. The ultimate strength of the films is 4.5 ± 0.2 GPa, and a comparison of the strength and pillar diameter with other non-nitride multi-component alloy materials is shown in Figure 5a, including single crystalline (SC) [100] CoCrCuFeNi multi-component alloy film pillar [27], NC Al_0.1_CoCrFeNi multi-component alloy film pillar [28], NC Al_0.3_CoCrFeNi multi-component alloy film pillar [29], SC [111] FeCoNiCuPd multi-component alloy film pillar [30]. The high strength can be associated with limited dislocation motion in the nanocrystalline materials with a high density of grain boundaries, hindering the movement of dislocations- the so-called Hall–Petch effect [31,32,33,34]. Meantime, the covalent bonds in the multi-component nitrides and the interstitial N in the multi-component alloys all contribute to enhancing the strength.

However, these values are not very prominent compared with other related nitride films. The sputtering methods and parameters greatly influence the density, microstructure, and quality of thin films, which may result in different hardness and strength, such as the coating environments, target power, voltage bias, temperature of the substrate and different deposition mechanisms [35,36,37,38,39,40]. The existence of non-covalent bonds in our films positively contributes to the ductility but may reduce the hardness simultaneously. High-quality multi-component alloy nitride films can be easily obtained by controlling the deposition parameters, but the growth mechanism and the influence of atomic-scale structures on the comprehensive mechanical properties may be more important. In summary, a schematic illustration of thin film’s microstructure and mechanical properties is presented in Figure 5b. Our work on the atomic-level microstructure and the mechanical performance of the nitride thin films may greatly enrich the implication potentials of multi-component alloys in the field of micro- and nano-devices.

## 4. Conclusions

This work fabricated the nanocrystalline structure (AlTiVCr)N multi-component nitride thin films with FCC structure by reactive magnetron sputtering. The atomic percent ratio value of N approaches more than 50 at.% due to the large electronegativity. Nanoscale columnar crystallites were observed by electron microscopy. XPS analysis suggested the coexistence of covalent and metallic bonds in the films. The excellent mechanical properties of the films are attributed to the synergistic effects of the nanoscale structure, covalent bonding between N and metal atoms, and interstitial strengthening.

## Data Availability

All data included in this study are available upon request by contact with the corresponding author.
